# Preservation of the Teeth

**Published:** 1860-05

**Authors:** J. Taft


### PRESERVATION OF THE TEETH.

Read before the Cincinnati Dental Association, at the regular meeting in February.

BY J. TAFT.

[Published by order of the Publishing Committee.]

THAT the utmost care and attention should be exercised for the health and well-being of the teeth, is not a question with any one who duly considers the matter. The well-being and health of the entire system is dependent, to a very considerable extent, upon the teeth. Very few seem to realize the value of these organs; particularly is this true in regard to the primary functions of the teeth. Many will express a strong desire that they may retain the teeth as organs of speech; frequently is the remark made, "I am very desirous of retaining my teeth, for they are valuable to me as a speaker." Others, again, appreciate them as ornaments. The remark is often made, "I would not lose my teeth, or have them injured for any consideration, for without teeth, one looks horridly." Now, while it is true the teeth are valuable in both these respects, they are more so in another, viz: mastication. While we would not in any case lessen the appreciation of them as organs of speech and beautifiers, we would have them far more highly prized as masticators.

The eye may become but slightly dimmed, or the ear but slightly dull, and alarm is at once created; the patient immediately betakes himself to those means and agencies which promise the most speedy and efficient relief. If anything is wrong, even in appearance, in either of these organs, it receives immediate attention. With the teeth, however, it is far otherwise; they may be in the most disgusting and unsightly condition, without calling forth the least anxiety on the part of their possessors; they may be invested with a deposition of foreign substances, that render them wholly incompatible with a refined *taste.*

In addition to this, the teeth may be wasting away by disease, which renders them still more unsightly, and yet the possessor exhibits no concern about it; they may even become so much diseased as to be sore and painful, and still, so long as this is not intolerable, they are permitted to pass along.

The means required for retaining the teeth in a healthy condition will be different in different cases. Various circumstances will modify the means that should be employed for their preservation; such as the health of the patient, the strength of vitality, the character of the secretions, the structure of the teeth, and the conditions of the contiguous soft tissues. While there is a system well developed in all its parts, all the different organs acting in harmony, and each one performing properly its functions, and possessing vigorous vitality, they all possess very nearly an equal resistance to injurious agencies, and hence, in a well-balanced economy in all particulars, the teeth would remain unimpaired, quite as long as the organs with which they are associated, as was evidently the design in the beginning. Such, however, is not the case, in the present condition of things. The teeth are often-times ruined, before complete development, and before the other organs have arrived at perfection.

It is proposed here briefly to refer to some of the conditions and causes of impairment of the teeth, and the manner of obviating the injury naturally accruing therefrom.

We propose, in the first place, to refer to those cases in which the teeth are good, and the conditions favorable. It might be supposed that, in such cases, the teeth would not become impaired; such, however, is not the result. Difficulty very frequently occurs with teeth of good structure, and surrounded by favorable systemic conditions. Impairment of such teeth may arise from two or three causes, all of which are extraneous,—none of which, perhaps, is more efficient than *a want of a proper amount of legitimate exercise.* Any organ when diverted from its *appropriate function,* or remains idle, becomes impaired in vitality, and consequently in ability to resist injurious influences ; hence, the dentine will be more readily attacked by disease, and the immediate soft tissues more subject to irritation.

The teeth from disease also suffer in another respect; they accumulate, and retain upon their surfaces, foreign matter, that is highly injurious to them. It is a habit very frequently contracted, of masticating all the food upon the teeth of one side ; sometimes it is purely a habit, in other cases, the person is forced to it by a diseased tooth ; but so far as a result is concerned, it is not material what is the cause. If the teeth are idle, they suffer from impaired vitality, and an accumulation and vitiation of extraneous substances upon them. The food may be of such a character as not to afford the teeth sufficient work to do, which is the case with the soft pultaceous food, so extensively used, which does not produce an adequate amount of friction and pressure upon the teeth. Both friction and pressure is necessary to keep them in a good condition : the former to prevent deposits of foreign substance upon them, the latter to invigorate the periosteal attachment. When a limb is not used, its muscles become shriveled and weak; when a tooth is not used, it loses vitality, the periosteum thickens, and the socket tends to obliteration, and the effort of nature is to discard the useless member.

The value of pressure and friction upon the teeth is exhibited in a very marked degree, by those who use tobacco. Such persons usually possess comparatively good teeth. (There is not in this, however, any apology for the filthy and disgusting habit of chewing tobacco.) This results from strengthened vitality by use quite as much, if not more than from the removal of foreign substance by the friction. The friction certainly is advantageous, particularly when it is of such character and upon such points, as to remove adhering substances ; some advantage is also attained, by the free bathing in the abundant flow of saliva, induced by the stimulus of the tobacco; but the great advantage gained by use is strengthening the vitality and attachment of the teeth.

The character of the food has much to do in giving due exercise to the teeth; it should be such as would afford some resistance in mastication. This is requisite, in order to give the teeth due exercise, and to produce adequate friction upon their surfaces.

The deposits upon the teeth differ very much in character and substance. There is the thick, gummy mucus, mixed up with food and foreign substances lodged upon the teeth, and passing through the various stages of putrefaction, affecting injuriously both the teeth and gums, where it may be in contact with the latter.

Deposits of this character are more pernicious than those of greater density; as they increase in density, and receive a larger proportion of calcareous ingredients, the liability to injury of the tooth tissue is correspondingly diminished; consequently, those deposits consisting almost solely of calcareous matter do not produce decomposition, or disease of the bone of the teeth.

The utmost care should be used for the removal of the deposits first referred to; this is very easily accomplished by the attention and well directed efforts of the patient. It can be done in most cases by the use of an appropriate tooth brush, but in other cases, a suitable dentifrice will be required. Such preparations should possess the following properties, viz: mechanical action sufficient to remove all deposits, alkalinity to neutralize acidity, and a de-odorizing property.

Ordinarily in a state of good health, the secretions normal, the proper kinds of food employed, the teeth would remain comparatively free from deposit of injurious substances, but so rarely do these conditions all exist in any given case, that the general rule is, that special care and attention is required to keep the teeth in a proper condition. It is true that the general health may to all appearance be good, and the teeth of good construction, and yet substances accumulate upon them, that will be highly injurious; this is particularly liable to occur, when a particular change takes place in the quality or relative quantity of the secretions of the mouth. An increased relative amount of mucus facilitates deposits upon the teeth; a vascid condition of the saliva is also productive of the same results. When such a condition exists, the utmost care is required, to secure cleanliness. The method of accomplishing this is in some sort familiar to all. When the secretions of the mouth have become changed, as just referred to, the preservation of the teeth calls for a restoration to their normal condition.

In order to accomplish this, a knowledge of physiology and pathology is required, for nothing short of this will enable us to accomplish the object with any certainty.

The causes of disease of the teeth are either general or local, or both; for the former, it is the business of the physician or scientific dentist to treat. The treatment of the latter is more the special business of the dentist, but the knowledge of the dentist should always be competent to detect these causes, wherever they may exist.

The preservation of the teeth, when they are influenced by general ill health, is exceedingly difficult. The particular treatment, especially the general treatment, would be modified and controlled by the condition. In all such cases, however, the utmost regard should be observed with reference to the treatment and removal of all local causes, for under such circumstances, local causes will produce greater injury.

In the preservation of the teeth, attention should be given to every thing that would in any way injuriously affect them. Sudden and extreme variations of temperature are injurious, or possess that tendency, but not to the *same* extent in all cases; some will resist such influences far more than others. The soft, highly organized teeth will be far more affected than the dense and less highly organized. While great care should be exercised by *all* persons in respect to changes of temperature, those having teeth highly susceptible to such influence, should be exceedingly cautious how they subject them to extremes of temperature.

Many things in which people so freely indulge, are very pernicious in this respect, such as hot coffee, tea, etc. These are used by many persons almost at boiling temperature ; this is attained by degrees ; to such an extent is this carried, that an uninitiated mouth would be scalded.

The teeth, as well as the mucous membrane, may accommodate themselves to *some extent* to this excess of temperature, but never can they endure it with impunity.

A very safe criterion in regard to the effect of changes of temperature upon the teeth, is found in those teeth that have not become accustomed to any unnatural condition. With such teeth excessive change of temperature, either by cold or heat, is attended with pain corresponding to the extent of the change.

Very cold fluids, as ice water, produce great pain in the teeth; habit will do much to enable the teeth to bear these transitions.

The greatest mischief is accomplished by the alternate use of hot and cold substances in immediate succession; the most intense pain may be produced in all the teeth in this way. It is not improbable but that necrosis is thus sometimes produced. But certainly in this way, the vitality of the teeth is much impaired.

Teeth in which there is a large relative amount of earthy elements, and consequently very friable, are lable to have the enamel checked by excessive changes of temperature, so much so as to facilitate the breaking away of portions of enamel; fractures facilitate caries.

In coffee, tea, soups, hot bread, *ice* water, soda water, etc., we find those things which are the vehicles of the greatest changes of temperature.

No other organs of the human system would endure abuse so great as this ; the eye, the ear, or any such organ, would be destroyed by far less powerful influences than many that are constantly operating upon the teeth. The teeth are organized with a view of withstanding and resisting injurious agencies; yet they can not endure all things. The teeth often suffer injury by being used for cracking or breaking hard substances, such as nuts and things of a similar character; lifting heavy weights, or drawing bodies of great resistance, gnashing the teeth, etc. I have attempted to enumerate some of the methods by which the teeth are injured; of course, for the preservation of the teeth; the indications are to refrain from practices leading to them, and in all cases, to promptly meet and counteract the unfavorable conditions.

### MODES OF PRACTICE AND THEIR VICISSITUDES.

BY J. TAFT.

